# Association of platelet count with incident motoric cognitive risk syndrome among Chinese adults aged 60 years or older: a national prospective cohort study

**DOI:** 10.1186/s12959-026-00908-y

**Published:** 2026-08-05

**Authors:** Na Yu, Xiaoyu Li, Qian Gui, Jing Jing, Qian Yu

**Affiliations:** 1 Department of Urology, The Second Affiliated Hospital of Dalian Medical University, Dalian, China; 2 School of Medicine, University of Electronic Science and Technology of China, Chengdu, China; 3 Wound Care Clinic, The Second Affiliated Hospital of Dalian Medical University, Dalian, China

**Keywords:** Platelet count, Motoric cognitive risk syndrome, Dementia, Neurodegeneration, Cohort study

## Abstract

**Background:**

Motoric cognitive risk syndrome (MCR), defined by subjective cognitive complaints and slow gait in individuals without dementia, is a clinically relevant pre-dementia phenotype. Platelets are increasingly linked to inflammation, endothelial dysfunction, and neurovascular signaling, but the prospective association between platelet count and MCR remains unclear.

**Objective:**

To examine whether baseline platelet count is associated with incident MCR among older Chinese adults.

**Methods:**

We analyzed 2,246 participants aged 60 years or older from the China Health and Retirement Longitudinal Study who were free of MCR at baseline in 2011 and had platelet count and follow-up MCR data in 2013. Platelet count was modeled as a continuous variable per 100 × 10⁹/L increase and by quintiles, with the middle quintile as the reference. Multivariable logistic regression models were adjusted for sociodemographic characteristics, lifestyle factors, adiposity indicators, depressive symptoms, and major chronic diseases. Restricted cubic spline analysis was used to evaluate the dose-response pattern. Subgroup analyses and a sensitivity analysis excluding participants with baseline stroke were also conducted.

**Results:**

During the 2-year follow-up, 264 participants (11.8%) developed MCR. In the fully adjusted model, each 100 × 10⁹/L increase in platelet count was associated with higher odds of incident MCR (odds ratio [OR] = 1.25, 95% confidence interval [CI]: 1.06–1.47; *P* = 0.007). Compared with the middle quintile, the fourth and fifth quintiles showed increased odds of MCR (Q4: OR = 1.62, 95% CI: 1.05–2.50; Q5: OR = 1.75, 95% CI: 1.14–2.69), whereas the lower quintiles were not statistically significant. Restricted cubic spline analysis indicated an overall association (*P* = 0.022) without clear evidence of non-linearity (*P* for non-linearity = 0.402). The association persisted after excluding participants with baseline stroke, and no statistically significant subgroup interactions were observed.

**Conclusions:**

Among Chinese adults aged 60 years or older, higher baseline platelet count was prospectively associated with increased odds of incident MCR. Platelet count may represent a readily measured hematologic correlate of incident MCR; however, the observational design precludes causal or predictive interpretation. Studies with longer follow-up and platelet activation, inflammatory, coagulation, and neuroimaging markers are needed to clarify the underlying mechanisms.

**Clinical trial number:**

Not applicable.

**Supplementary Information:**

The online version contains supplementary material available at https://doi.org/10.1186/s12959-026-00908-y.

## Introduction

Platelets have long been regarded as the primary effector cells of the hemostatic system; however, accumulating evidence demonstrates that their biological functions extend far beyond this traditional role [1–3]. Platelets express various receptors for neuroactive substances on their surface and store and release neuromodulatory molecules such as serotonin, glutamate, and brain-derived neurotrophic factor [4, 5]. Notably, platelets represent one of the major peripheral sources of amyloid precursor protein (APP) and its metabolite β-amyloid (Aβ), which can be transported across the blood-brain barrier to participate in central nervous system pathological processes [6, 7]. Clinical studies have found that patients with Alzheimer’s disease exhibit abnormal peripheral platelet activation states, and platelet count shows significant correlations with cognitive function scores [8, 9]. These findings suggest that platelets may serve as an important mediator linking the peripheral blood system with central neurodegenerative changes, and alterations in platelet count may function as a potential biomarker reflecting brain health status.

Motoric Cognitive Risk Syndrome (MCR), a novel pre-dementia syndrome proposed by Verghese and colleagues in 2013, is characterized by the coexistence of objectively measured slow gait and subjective cognitive complaints without meeting diagnostic criteria for dementia [10]. Compared to mild cognitive impairment (MCI), MCR diagnosis does not require complex neuropsychological assessments, making it more suitable for large-scale community screening [11, 12]. International multicenter cohort studies have confirmed that individuals with MCR have nearly twice the risk of developing dementia compared to those without MCR, and MCR is independently associated with risks of mortality, falls, and disability [13–15]. As one of the countries with the fastest aging population globally, China has an MCR prevalence of approximately 9.74%-12.8% [16–18], highlighting the importance of identifying simple and accessible biomarkers that may help detect individuals at elevated risk of MCR.

Currently, the association between platelet count and MCR incidence remains unclear. Previous studies exploring the relationship between blood markers and cognitive function have predominantly employed cross-sectional designs, which limit causal inference, and some have used linear analytical approaches that may overlook potential non-linear effects [19, 20]. Therefore, we used prospective cohort data from the China Health and Retirement Longitudinal Study (CHARLS) spanning 2011–2013 to examine the association between baseline platelet count and incident MCR and to explore its possible mechanistic relevance.

## Methods

### Study design and study population

**Fig. 1:**
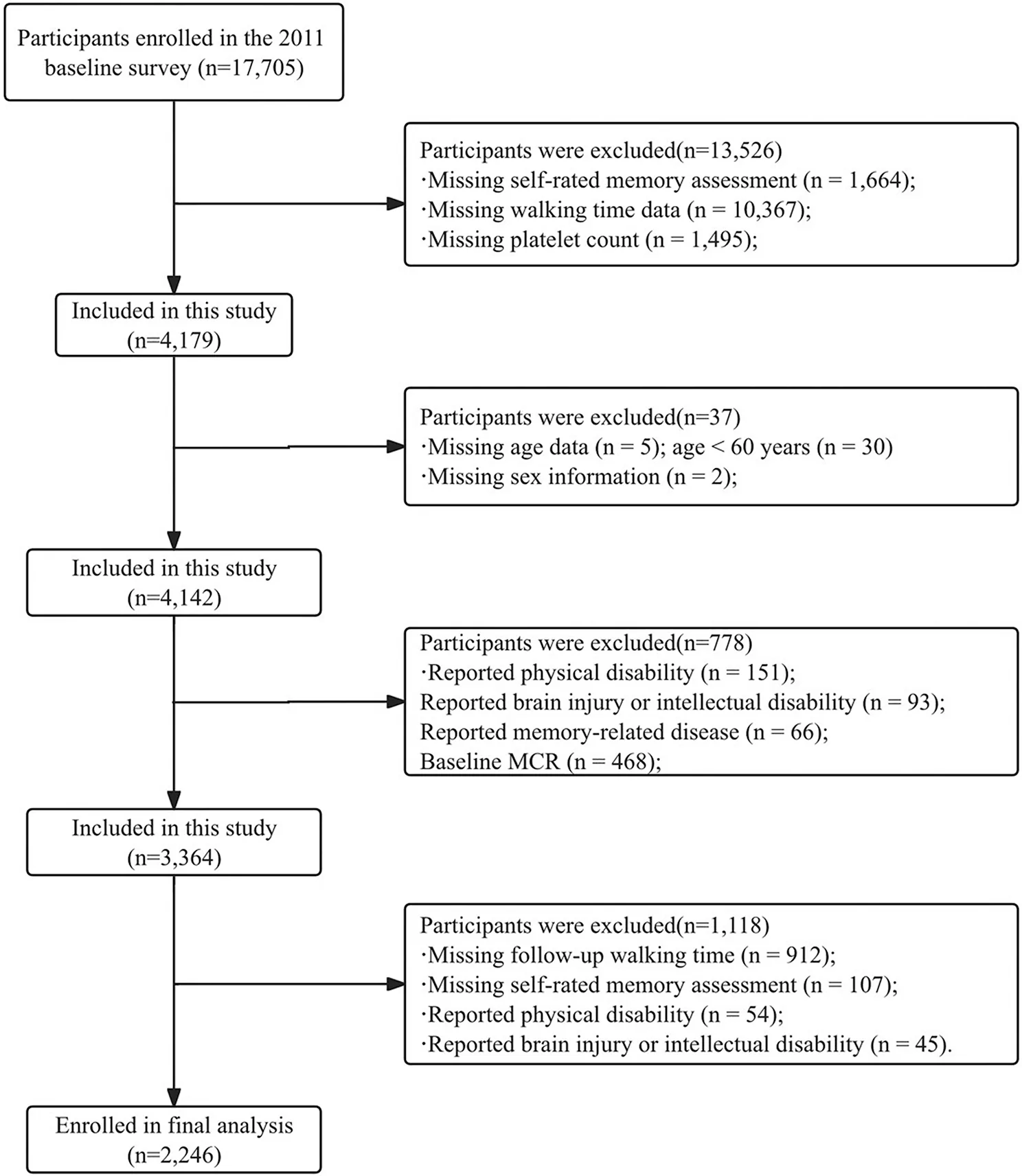
Flowchart depicting the screening and enrollment of subjects

This prospective cohort study utilized data from the China Health and Retirement Longitudinal Study (CHARLS). CHARLS employed a multistage stratified cluster sampling approach, covering 450 villages across 150 counties in 28 provinces nationwide, ensuring strong national representativeness [21]. This study used the 2011 baseline survey as the exposure assessment timepoint and the 2013 follow-up survey as the outcome observation endpoint. The study protocol was approved by the Biomedical Ethics Committee of Peking University (approval number: IRB00001052-11015), and all participants provided written informed consent. Inclusion criteria were: (1) age ≥ 60 years; (2) completion of venous blood platelet count testing; and (3) availability of complete gait speed measurements and cognitive complaint assessment data. A total of 2,246 respondents were ultimately included, as shown in Fig. 1. Participants with MCR at baseline were excluded; therefore, individuals free of MCR at baseline constituted the internal reference population for evaluating incident MCR during follow-up. As this was a population-based cohort rather than a case-control study, no separately defined healthy control group was established.

### Exposure variable

Platelet count (×10⁹/L) was obtained from the CHARLS 2011 baseline complete blood count data. Venous blood samples were collected by medically trained China CDC staff according to a standardized protocol. Participants were asked to fast overnight, and fasting status was recorded. The first 2-mL blood tube was used for complete blood count testing, including platelet count, which was performed using automated analyzers at local CDC stations or township/village health centers. Fresh samples were transported at 4 °C, and for most fasting specimens collected at centralized sites, complete blood count testing was completed within 141 min of collection, with a median collection-to-assay time of 97 min. More than 97% of participating laboratories were enrolled in regular assay quality-control programs [22]. Platelet count was modeled as a continuous variable for non-linear analysis and categorized into quintiles for categorical analysis.

### Outcome variable

MCR diagnosis followed the criteria proposed by Verghese et al. and was defined as the coexistence of slow gait and subjective cognitive complaint in participants without dementia, brain damage, intellectual disability, memory-related disease, or physical disability [23]. Gait speed was calculated based on two 2.5-meter walking tests, and the average gait speed was used in the analysis. Slow gait was defined using sex- and age-specific reference values. Specifically, participants were stratified by sex and 5-year age groups, with those aged 80 years and older classified as a separate group; within each stratum, slow gait was defined as gait speed at least one standard deviation below the corresponding mean.

Subjective cognitive complaint was assessed using CHARLS item DC004, which asked: “How would you rate your current memory? Excellent, very good, good, fair, poor, or I don’t know?” Participants who responded “fair” or “poor” were classified as having subjective cognitive complaint. Previous analyses of CHARLS data have supported the reliability of self-rated memory in population studies [24, 25]. Participants with physical disability, brain damage, intellectual disability, or memory-related disease were excluded using CHARLS items DA005_1, DA005_2, and DA007_12. DA005 asked: “Do you have one of the following disabilities?” Physical disability was identified using DA005_1, and brain damage or mental retardation, as worded in the original CHARLS questionnaire, was identified using DA005_2. Memory-related disease was identified using DA007_12 from DA007, which asked: “Have you been diagnosed with the following conditions by a doctor?” This item referred to memory-related diseases, including Alzheimer’s disease, brain atrophy, and Parkinson’s disease. Incident MCR in 2013 was defined as not meeting the MCR criteria at baseline but fulfilling all criteria at follow-up.

### Covariates

Covariates were selected based on previous literature and clinical relevance, including: sociodemographic characteristics (age, sex, years of education, marital status, household registration status); health behaviors (current smoking, alcohol consumption in the past year); standardized measurements of waist circumference and BMI; chronic diseases (hypertension, diabetes, lung disease, heart disease, and stroke), defined based on self-reported physician diagnoses and, where available, self-reported use of related medications; and mental health, assessed using the Center for Epidemiologic Studies Depression Scale (CES-D) score.

### Statistical analysis

For baseline characteristic description, participants were categorized according to incident MCR status during follow-up: the non-MCR group and the MCR group. Baseline sociodemographic characteristics, lifestyle factors, anthropometric indicators, hematological parameters, and comorbidities were compared between the two groups. For association analyses, participants were divided into five groups based on platelet count quintiles (Q1-Q5). Normality of continuous variables was assessed using the Shapiro-Wilk test. Normally distributed variables were presented as mean ± standard deviation and compared using one-way analysis of variance. Non-normally distributed variables were presented as median with interquartile range and compared using the Kruskal-Wallis H test. Categorical variables were presented as frequencies and percentages and compared using chi-square test or Fisher’s exact test.

Restricted cubic spline (RCS) logistic regression was used to model the dose-response relationship between platelet count and incident MCR [26]. Missing covariates were handled using multiple imputation (5 iterations) via the mice package in R, with predictive mean matching for continuous variables and logistic regression for binary variables; results were pooled according to Rubin’s rules. Exploratory subgroup analyses were performed to assess potential effect heterogeneity across clinically relevant strata, including age, sex, smoking status, alcohol consumption, hypertension, diabetes, and heart disease. In these analyses, platelet count was modeled as a continuous variable scaled per 100 × 10⁹/L increase to improve interpretability and model stability. Education level and baseline stroke were not included as subgroup variables because of the limited number of incident MCR events in some strata. Effect modification was assessed by including multiplicative interaction terms between platelet count and each subgroup variable. Given the number of subgroup and interaction analyses, these results were considered exploratory and interpreted cautiously. All statistical analyses were performed using R software version 4.3.0, with two-sided *P* < 0.05 considered statistically significant. Because only 40 participants reported baseline stroke, the stroke-exclusion analysis was treated as an exploratory sensitivity check rather than a definitive subgroup analysis.

## Results

### Baseline characteristics of study participants

A total of 2,246 participants were included in the analysis, of whom 264 developed incident motoric cognitive risk syndrome (MCR) during follow-up (Table 1). Compared with participants without MCR, those who developed MCR had a significantly lower level of educational attainment (*P* = 0.002), higher depressive symptom scores as assessed by the CES-D scale (10.9 ± 5.7 vs. 9.6 ± 4.7, *P* < 0.001), higher platelet counts (221.0 ± 89.7 vs. 206.6 ± 72.9 × 10⁹/L, *P* = 0.003), and a greater prevalence of prior stroke (4.5% vs. 1.4%, *P* = 0.001). No statistically significant differences were observed between groups in age, sex, hukou status, marital status, BMI, waist circumference, smoking status, alcohol consumption, white blood cell count, hemoglobin, hematocrit, or major chronic conditions including hypertension, diabetes, lung disease, and heart disease.

**Table 1:** Baseline characteristics of participants according to incident motoric cognitive risk syndrome status

Variables	Total (*n* = 2246)	No MCR (*n* = 1982)	MCR (*n* = 264)	*P*
Age, Mean ± SD	67.1 ± 5.9	67.1 ± 5.8	67.5 ± 6.1	0.209
Sex, n (%)				0.195
Female	1124 (50.0)	982 (49.5)	142 (53.8)	
Male	1122 (50.0)	1000 (50.5)	122 (46.2)	
Hukou status, n (%)				0.063
Agricultural	1857 (82.7)	1628 (82.1)	229 (86.7)	
Other	389 (17.3)	354 (17.9)	35 (13.3)	
Education, n (%)				0.002
Below primary school	1267 (56.4)	1102 (55.6)	165 (62.5)	
Primary school	606 (27.0)	529 (26.7)	77 (29.2)	
Middle school	256 (11.4)	239 (12.1)	17 (6.4)	
High school and above	117 (5.2)	112 (5.7)	5 (1.9)	
Marital status, n (%)				0.06
Married	1807 (80.5)	1606 (81)	201 (76.1)	
Other	439 (19.5)	376 (19)	63 (23.9)	
BMI ^a^, Mean ± SD	22.9 ± 3.8	22.9 ± 3.7	22.7 ± 4.4	0.57
CESD, Mean ± SD	9.8 ± 4.9	9.6 ± 4.7	10.9 ± 5.7	< 0.001
WC ^a^, Mean ± SD	83.8 ± 12.6	83.8 ± 12.5	83.6 ± 13.4	0.852
WBC, Mean ± SD	6.2 ± 1.9	6.2 ± 1.9	6.4 ± 1.9	0.23
MCV, Mean ± SD	91.2 ± 8.5	91.1 ± 8.5	91.7 ± 8.6	0.319
PLT, Mean ± SD	208.3 ± 75.2	206.6 ± 72.9	221.0 ± 89.7	0.003
HGB, Mean ± SD	14.3 ± 2.2	14.3 ± 2.2	14.3 ± 2.1	0.983
HCT, Mean ± SD	41.3 ± 6.3	41.4 ± 6.3	40.6 ± 6.4	0.069
Smoke, n (%)				0.799
No	1301 (57.9)	1150 (58)	151 (57.2)	
Yes	945 (42.1)	832 (42)	113 (42.8)	
Drink ^a^, n (%)				0.34
No	1673 (74.5)	1470 (74.2)	203 (76.9)	
Yes	573 (25.5)	512 (25.8)	61 (23.1)	
Hypertension ^a^, n (%)				0.478
No	1607 (71.5)	1423 (71.8)	184 (69.7)	
Yes	639 (28.5)	559 (28.2)	80 (30.3)	
Diabetes ^a^, n (%)				0.333
No	1894 (84.3)	1666 (84.1)	228 (86.4)	
Yes	352 (15.7)	316 (15.9)	36 (13.6)	
Lung disease ^a^, n (%)				0.913
No	1953 (87.0)	1724 (87)	229 (86.7)	
Yes	293 (13.0)	258 (13)	35 (13.3)	
Heart disease ^a^, n (%)				0.28
No	1937 (86.2)	1715 (86.5)	222 (84.1)	
Yes	309 (13.8)	267 (13.5)	42 (15.9)	
Stroke ^a^, n (%)				0.001
No	2206 (98.2)	1954 (98.6)	252 (95.5)	
Yes	40 (1.8)	28 (1.4)	12 (4.5)	

Notes: a Missing data: 14 for BMI; 4 for WC; 477 for Drink; 8 for Hypertension; 12 for Diabetes; 6 for lung diseases; 13 for Heart disease; 5 for Stroke

Abbreviations: SD, standard deviation; IQR, interquartile range; BMI, body mass index; CES-D, Center for Epidemiologic Studies Depression Scale; WC, waist circumference; WBC, white blood cell count; MCV, mean corpuscular volume; PLT, platelet count; HGB, hemoglobin; HCT, hematocrit; MCR, motoric cognitive risk syndrome

### Association between platelet count and incident MCR

Multivariable logistic regression analyses showed that platelet count was associated with incident motoric cognitive risk syndrome (MCR) across all models (Table 2). When platelet count was modeled as a continuous variable scaled per 100 × 10⁹/L increase, higher platelet count was consistently associated with greater odds of incident MCR. This association remained statistically significant after progressive adjustment for demographic characteristics, lifestyle factors, adiposity-related indicators, depressive symptoms, and major chronic diseases. In the fully adjusted model, each 100 × 10⁹/L increase in platelet count was associated with a 25% higher odds of incident MCR (OR = 1.25, 95% CI: 1.06–1.47, *P* = 0.007).

Using the third quintile of platelet count as the reference group, participants in the fourth and fifth quintiles had significantly increased odds of incident MCR. In the fully adjusted model, the ORs were 1.62 (95% CI: 1.05–2.50, *P* = 0.028) for Q4 and 1.75 (95% CI: 1.14–2.69, *P* = 0.010) for Q5. In contrast, the associations for Q1 and Q2 were not statistically significant. The trend test did not reach statistical significance in the fully adjusted model (OR = 1.08, 95% CI: 0.98–1.18, *P* = 0.129), suggesting that the elevated odds of MCR were mainly observed among participants with higher platelet counts rather than following a strictly linear dose-response pattern.

**Table 2:** Multivariable logistic regression analysis of the association between platelet count and incident motoric cognitive risk syndrome

Variable	Model 1		Model 2		Model 3		Model 4	
	OR (95%CI)	*P* value	OR (95%CI)	*P* value	OR (95%CI)	*P* value	OR (95%CI)	*P* value
PLT	1.27 (1.08 ~ 1.49)	0.004	1.26 (1.07 ~ 1.48)	0.005	1.24 (1.06 ~ 1.46)	0.008	1.25 (1.06 ~ 1.47)	0.007
Q3	1(Ref)		1(Ref)		1(Ref)		1(Ref)	
Q1	1.38 (0.89 ~ 2.15)	0.146	1.37 (0.88 ~ 2.14)	0.16	1.42 (0.91 ~ 2.21)	0.125	1.39 (0.89 ~ 2.17)	0.151
Q2	1.3 (0.84 ~ 2.02)	0.245	1.3 (0.83 ~ 2.03)	0.245	1.32 (0.85 ~ 2.07)	0.221	1.33 (0.85 ~ 2.08)	0.214
Q4	1.62 (1.06 ~ 2.49)	0.026	1.6 (1.04 ~ 2.46)	0.031	1.63 (1.06 ~ 2.51)	0.025	1.62 (1.05 ~ 2.5)	0.028
Q5	1.79 (1.18 ~ 2.73)	0.007	1.75 (1.15 ~ 2.67)	0.01	1.76 (1.15 ~ 2.69)	0.009	1.75 (1.14 ~ 2.69)	0.01
Trend test	1.08 (0.99 ~ 1.19)	0.088	1.08 (0.98 ~ 1.18)	0.115	1.07 (0.98 ~ 1.18)	0.139	1.08 (0.98 ~ 1.18)	0.129

Abbreviations: PLT, platelet count; Q, quintile; OR, odds ratio; CI, confidence interval; Ref, reference

Model 1: Unadjusted crude model

Model 2: Adjusted for age, sex, Hukou, educational level, and marital status

Model 3: Adjusted for covariates in Model 2 plus Body Mass Index (BMI), waist circumference, smoking status, alcohol consumption, and depressive symptoms (CES-D score)

Model 4: Adjusted for covariates in Model 3 plus history of hypertension, diabetes, heart disease, chronic lung disease, and stroke

### Restricted cubic spline analysis of the dose–response association

**Fig. 2:**
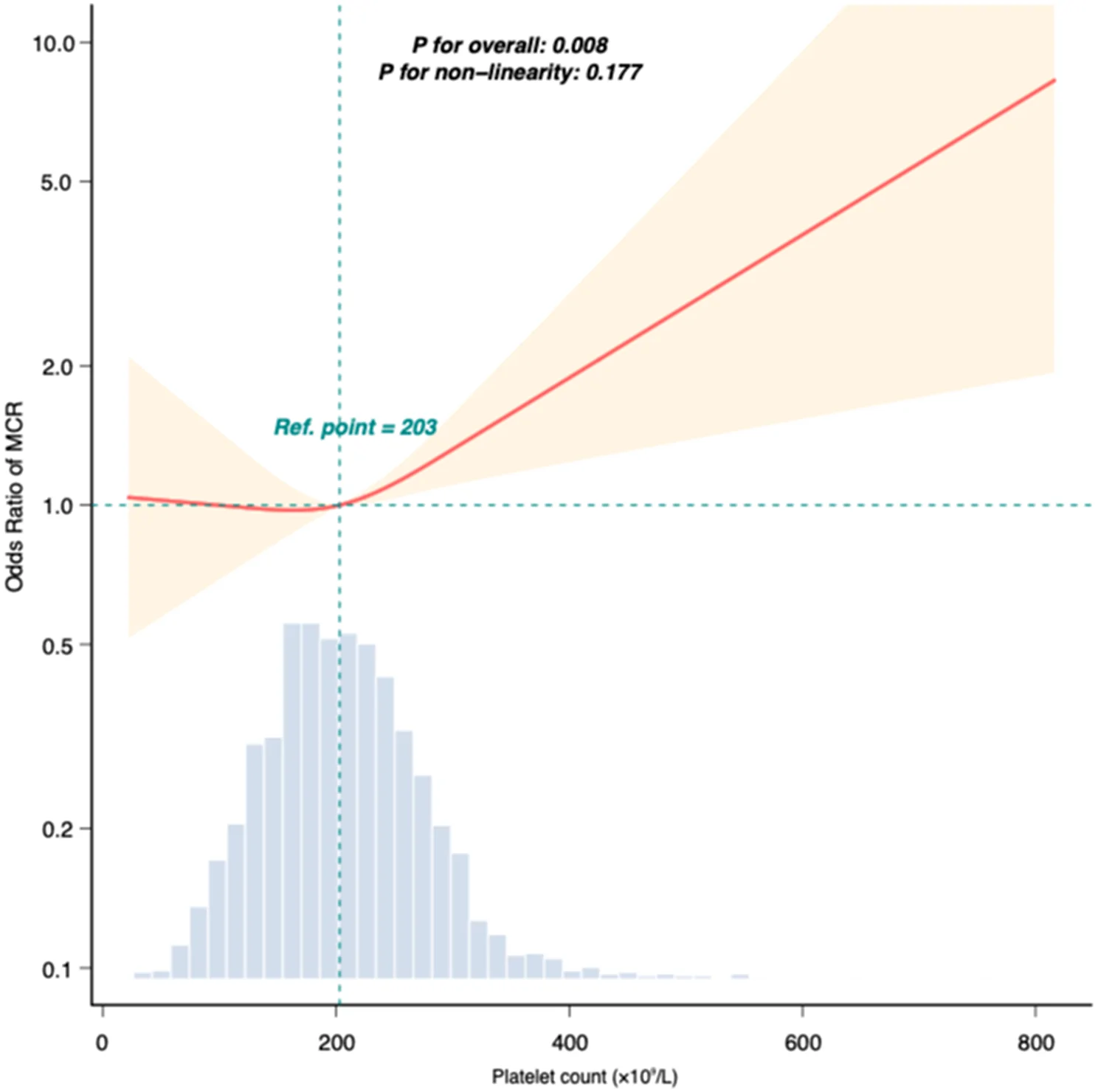
Association between baseline platelet count and incident MCR estimated using restricted cubic spline logistic regression. The solid red line shows the adjusted odds ratio (OR), and the shaded area shows the 95% confidence interval. Dashed lines mark the reference platelet count of 203 × 10⁹/L and OR of 1.0; the bars show the distribution of platelet counts. Platelet count is expressed in × 10⁹/L. P values for the overall association and non-linearity are shown in the figure. Estimates at the extremes of the platelet-count distribution are imprecise because data are sparse and should be interpreted cautiously

Restricted cubic spline logistic regression was performed to further assess the dose-response relationship between platelet count and incident MCR after adjustment for sociodemographic characteristics, lifestyle factors, adiposity, depressive symptoms, and major chronic conditions. As shown in Fig. 2, platelet count was significantly associated with incident MCR in the overall association test (*P* for overall association = 0.022). However, the test for non-linearity was not statistically significant (*P* for non-linearity = 0.402), indicating no clear evidence of a non-linear dose-response pattern. Overall, the RCS analysis suggested that higher platelet count was associated with increased odds of incident MCR.

### Subgroup effect evaluation of the association between platelet count and MCR

**Fig. 3:**
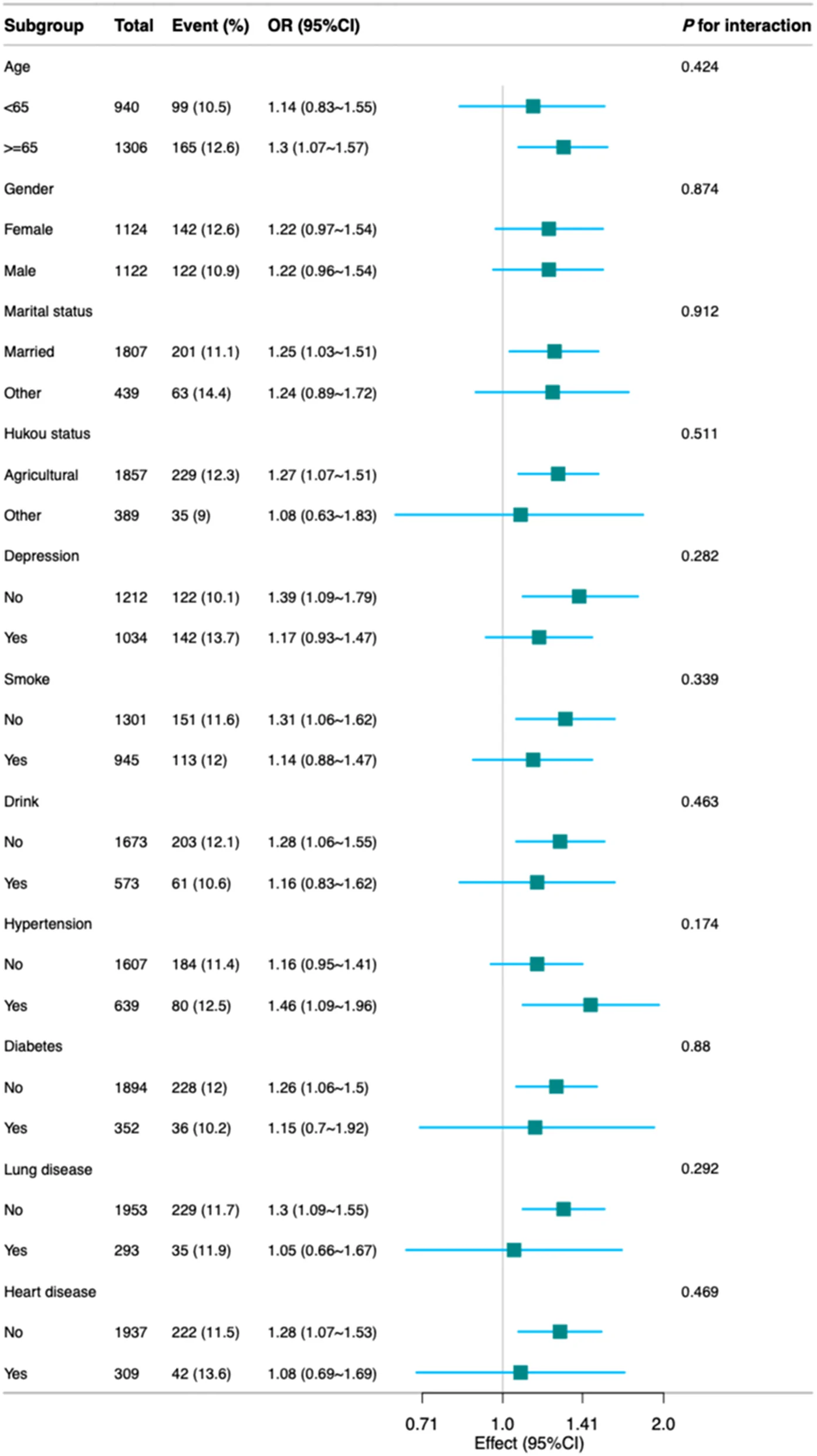
Subgroup associations between baseline platelet count and incident MCR. Squares show odds ratios (ORs) for each 100 × 10⁹/L increase in platelet count, horizontal lines show 95% confidence intervals, and P values are for interaction tests. The vertical line denotes OR = 1.0. Subgroup estimates are exploratory and should be interpreted cautiously

Exploratory subgroup analyses were performed to evaluate potential effect modification across clinically relevant strata. Platelet count was modeled as a continuous variable scaled per 100 × 10⁹/L increase. As shown in Fig. 3, the direction of association was generally consistent across subgroups, with most point estimates greater than 1.0. No statistically significant interactions were observed for age, sex, marital status, household registration status, depression status, smoking, alcohol consumption, hypertension, diabetes, lung disease, or heart disease, suggesting no clear evidence of effect modification.

### Sensitivity analysis

To evaluate the potential influence of baseline stroke on the association between platelet count and incident MCR, we excluded participants with baseline stroke and repeated the primary analyses. A total of 40 participants (1.8%) had baseline stroke, including 12 who developed incident MCR (Table 1); the stroke-exclusion analysis was therefore treated as an exploratory robustness check rather than a definitive subgroup comparison. After excluding these 40 participants, platelet count modeled as a continuous variable remained associated with incident MCR across all models. In the fully adjusted model, each 100 × 10⁹/L increase in platelet count was associated with higher odds of incident MCR (OR = 1.28, 95% CI: 1.08–1.50, *P* = 0.004).

When platelet count was analyzed by quintiles, the associations for the fourth and fifth quintiles remained statistically significant compared with the middle quintile. In the fully adjusted model, the ORs were 1.67 (95% CI: 1.07–2.60, *P* = 0.024) for Q4 and 1.86 (95% CI: 1.20–2.88, *P* = 0.005) for Q5. By contrast, the estimates for Q1 and Q2 were not statistically significant. The trend test was also not statistically significant in the fully adjusted model (OR = 1.09, 95% CI: 0.99–1.19, *P* = 0.094). These findings suggest that the positive association between higher platelet count and incident MCR persisted after excluding participants with baseline stroke; however, given the small baseline-stroke group, this analysis should be viewed as a robustness check rather than independent evidence.

## Discussion

In this national prospective cohort of older Chinese adults, baseline platelet count was associated with incident MCR over a 2-year follow-up. After adjustment for demographic characteristics, lifestyle factors, adiposity, depressive symptoms, and major chronic conditions, higher platelet counts were consistently associated with increased odds of incident MCR. This association remained evident after excluding participants with baseline stroke. In contrast, lower platelet count categories were not clearly associated with incident MCR in the fully adjusted and sensitivity analyses. These findings suggest that platelet count, particularly at higher levels, may be a readily measured hematologic correlate of incident MCR. Given the observational design, however, the results should be interpreted as associations rather than evidence of causality or predictive utility. Notably, the high proportion of participants with lower educational attainment is consistent with the older and predominantly agricultural CHARLS population and should be interpreted within the demographic and socioeconomic context of the source cohort.

These findings are biologically plausible in light of growing evidence that platelets are involved in processes beyond hemostasis. Platelets participate in inflammation, endothelial regulation, immune signaling, and neurovascular interactions [27–30]. They also store and release neuroactive molecules, including serotonin, glutamate, and brain-derived neurotrophic factor, and have been linked to amyloid precursor protein and β-amyloid metabolism [5, 7]. In Alzheimer’s disease and related neurodegenerative disorders, platelet abnormalities have more often been discussed in terms of platelet activation, platelet function, and platelet heterogeneity than platelet quantity alone [31–33]. Therefore, platelet count should not be interpreted as a direct measure of platelet dysfunction. Rather, it may represent a readily available but indirect correlate of platelet-related biological status in population-based studies.

The association observed at higher platelet counts may partly reflect thrombo-inflammatory and microvascular pathways. Elevated platelet count may indicate a prothrombotic or proinflammatory state, characterized by enhanced platelet-endothelial interactions, release of inflammatory mediators, and activation of coagulation-related pathways [34, 35]. These processes may contribute to endothelial dysfunction, impaired cerebral microcirculation, and cerebral small vessel injury. Previous studies have linked platelet-related or systemic inflammatory markers with cerebral small vessel disease and white matter hyperintensity burden [36, 37]. Such microvascular changes may disrupt frontal-subcortical networks involved in gait regulation and cognitive processing, providing a plausible pathway through which higher platelet count could be associated with MCR [38]. This interpretation is consistent with the clinical construct of MCR, which combines slow gait and subjective cognitive complaint and has been associated with both vascular and neurodegenerative risk.

Several limitations should be considered when interpreting these findings. First, although we adjusted for demographic characteristics, lifestyle factors, adiposity, depressive symptoms, and major chronic diseases, residual confounding cannot be excluded. Platelet count may be influenced by inflammation, hematologic disorders, disease severity, nutritional status, medication use, and other clinical conditions that were not fully captured in CHARLS. In particular, detailed information on antiplatelet agents, anticoagulants, corticosteroids, anti-inflammatory drugs, chemotherapy, medication dose, treatment duration, and indications was unavailable, limiting our ability to distinguish disease-related from medication-related platelet variability. Second, platelet count was measured only once at baseline, and we could not assess long-term platelet trajectories or transient changes related to infection, inflammation, or other short-term conditions. Third, CHARLS did not provide platelet function or activation markers, such as mean platelet volume, platelet distribution width, plateletcrit, platelet activation markers, or coagulation-related biomarkers. Therefore, we could not directly evaluate platelet activation, platelet heterogeneity, or thrombo-inflammatory pathways. Fourth, MCR ascertainment relied partly on a self-reported memory complaint and self-reported exclusion conditions, including memory-related disease; chronic disease diagnoses and medication use were also self-reported. Recall or reporting error may therefore have caused outcome or covariate misclassification. Fifth, only 40 participants had baseline stroke and 12 of these developed incident MCR; consequently, the stroke-exclusion sensitivity analysis should be interpreted as an exploratory robustness check. Sixth, the follow-up period was relatively short. Although the prospective design supports temporal ordering between baseline platelet count and incident MCR, longer follow-up is needed to evaluate the long-term relationship between platelet-related changes and cognitive-motor decline.

Despite these limitations, this study has several strengths. It used data from a large, nationally representative prospective cohort, excluded participants with MCR at baseline, and applied multivariable models and restricted cubic spline analyses to examine the dose-response pattern. Platelet count is routinely measured, inexpensive, and widely available; however, the present findings support its interpretation only as a readily measured correlate of incident MCR and do not establish screening or predictive utility. Future studies incorporating platelet activation markers, inflammatory and coagulation profiles, neuroimaging measures, and longer follow-up are needed to clarify the biological pathways underlying this association.

## Conclusion

In summary, this national prospective cohort study found that higher baseline platelet count was associated with increased odds of incident MCR among Chinese adults aged 60 years and older. The observed association may partly reflect platelet-related thrombo-inflammatory or microvascular processes, although the present study cannot establish causality, predictive performance, or clinical utility and cannot directly assess platelet activation pathways. Platelet count should therefore be regarded as a readily measured correlate that warrants further mechanistic and longitudinal investigation rather than as a validated risk-prediction or early-intervention tool. Future studies incorporating detailed platelet function markers, inflammatory and coagulation profiles, neuroimaging data, and longer follow-up are warranted to validate these findings and clarify the underlying mechanisms.

## Supplementary Information

Below is the link to the electronic supplementary material.

Supplementary Material 1

## Data Availability

The datasets analyzed during the current study are publicly available in the China Health and Retirement Longitudinal Study (CHARLS) repository (http://charls.pku.edu.cn/en).
