# Supplementary material for: Association of platelet count with incident motoric cognitive risk syndrome among Chinese adults aged 60 years or older: a national prospective cohort study
Source: Thromb J. 2026 Aug 5;24:102. doi: 10.1186/s12959-026-00908-y (PMC13726130; doi:10.1186/s12959-026-00908-y)
Supplement: Supplementary file 1 [file 12959_2026_908_MOESM1_ESM.docx]

**Supplementary Material for:**

Association Between Platelet Count and Incident Motoric Cognitive Risk Syndrome in Older Adults: A National Prospective Cohort Study

**Contents:**

Supplementary Table 1: Multivariable logistic regression analysis of the association between Platelet and Motoric Cognitive Risk Syndrome (MCR) among participants without anemia.

**Supplementary Table 1.** Sensitivity Analysis: Association Between Platelet and Motoric Cognitive Risk Syndrome After Excluding Participants with Stroke

| **Variable** | **Model 1** | | **Model 2** | | **Model 3** | | **Model 4** | |
| --- | --- | --- | --- | --- | --- | --- | --- | --- |
|  | **OR (95%CI)** | **P value** | **OR (95%CI)** | **P value** | **OR (95%CI)** | **P value** | **OR (95%CI)** | **P value** |
| PLT | 1.29 (1.1~1.52) | 0.002 | 1.29 (1.1~1.52) | 0.002 | 1.27 (1.08~1.5) | 0.004 | 1.28 (1.08~1.5) | 0.004 |
| Q3 | 1(Ref) |  | 1(Ref) |  | 1(Ref) |  | 1(Ref) |  |
| Q1 | 1.38 (0.88~2.18) | 0.163 | 1.36 (0.86~2.15) | 0.192 | 1.4 (0.88~2.22) | 0.155 | 1.38 (0.87~2.19) | 0.173 |
| Q2 | 1.42 (0.9~2.23) | 0.131 | 1.41 (0.89~2.22) | 0.14 | 1.43 (0.9~2.25) | 0.126 | 1.43 (0.91~2.26) | 0.125 |
| Q4 | 1.67 (1.08~2.6) | 0.022 | 1.65 (1.06~2.56) | 0.027 | 1.68 (1.08~2.62) | 0.022 | 1.67 (1.07~2.6) | 0.024 |
| Q5 | 1.89 (1.23~2.91) | 0.004 | 1.84 (1.19~2.85) | 0.006 | 1.86 (1.2~2.88) | 0.005 | 1.86 (1.2~2.88) | 0.005 |
| Trend test | 1.09 (0.99~1.2) | 0.074 | 1.09 (0.99~1.19) | 0.085 | 1.08 (0.98~1.19) | 0.1 | 1.09 (0.99~1.19) | 0.094 |

Abbreviations: PLT, platelet count; Q, quintile; OR, odds ratio; CI, confidence interval; Ref, reference.

**Model 1:** Unadjusted crude model.

**Model 2:** Adjusted for age, sex, Hukou, educational level, and marital status.

**Model 3:** Adjusted for covariates in Model 2 plus Body Mass Index (BMI), waist circumference, smoking status, alcohol consumption, and depressive symptoms (CES-D score).

**Model 4:** Adjusted for covariates in Model 3 plus history of hypertension, diabetes, heart disease, and chronic lung disease.
